# Coordinated regulation of photosynthetic and respiratory components is necessary to maintain chloroplast energy balance in varied growth conditions

**DOI:** 10.1093/jxb/erw469

**Published:** 2016-12-23

**Authors:** Keshav Dahal, Greg D. Martyn, Nicole A. Alber, Greg C. Vanlerberghe

**Affiliations:** 1 Department of Biological Sciences and Department of Cell and Systems Biology, University of Toronto Scarborough, 1265 Military Trail, Toronto, ON M1C1A4,Canada

**Keywords:** Chloroplast energy balance, drought stress, excitation pressure, growth irradiance, light-harvesting complex II protein, mitochondrial alternative oxidase, photosynthesis, respiration.

## Abstract

Mitochondria have a non-energy-conserving alternative oxidase (AOX) proposed to support photosynthesis, perhaps by promoting energy balance under varying growth conditions. To investigate this, wild-type (WT) *Nicotiana tabacum* were compared with AOX knockdown and overexpression lines. In addition, the amount of AOX protein in WT plants was compared with that of chloroplast light-harvesting complex II (LHCB2), whose amount is known to respond to chloroplast energy status. With increased growth irradiance, WT leaves maintained higher rates of respiration in the light (*R*_L_), but no differences in *R*_L_ or photosynthesis were seen between the WT and transgenic lines, suggesting that, under non-stress conditions, AOX was not critical for leaf metabolism, regardless of growth irradiance. However, under drought, the AOX amount became an important determinant of *R*_L_, which in turn was an important determinant of chloroplast energy balance (measured as photosystem II excitation pressure, EP), and photosynthetic performance. In the WT, the AOX amount increased and the LHCB2 amount decreased with increased growth irradiance or drought severity. These changes in protein amounts correlated strongly, in opposing ways, with growth EP. This suggests that a signal deriving from the photosynthetic electron transport chain status coordinately controls the amounts of AOX and LHCB2, which then both contribute to maintaining chloroplast energy balance, particularly under stress conditions.

## Highlight

Plants use the alternative oxidase to modulate the rate of mitochondrial respiration in the light, maintaining chloroplast energy balance and hence optimizing photosynthesis under varied growth conditions.

## Introduction

Respiration in the mitochondrion and photosynthesis in the chloroplast share important carbon and energy intermediates, and hence it is thought that these pathways must act in a coordinated manner to optimize energy metabolism in the leaf cell (Hoefnagel *et al.*, 1998; Gardestrӧm *et al.*, 2002; Noctor *et al.*, 2007; Noguchi and Yoshida, 2008; Nunes-Nesi *et al.*, 2008; Tcherkez *et al.*, 2012; Gardestrӧm and Igamberdiev, 2016). For example, an imbalance of energy intermediates in one organelle might be offset by compensatory metabolic changes in the other organelle. Numerous studies have investigated the potential role of the non-energy-conserving alternative oxidase (AOX) pathway of mitochondrial electron transport in optimizing photosynthesis (see below). Unlike electron flow from ubiquinol to cytochrome oxidase, electron flow from ubiquinol to AOX is not proton-pumping and hence is not limited by the rate of ATP turnover. The maximum capacity of the AOX pathway to consume electrons is dependent upon developmental and environmental cues, and the actual partitioning of electrons to AOX is subject to sophisticated biochemical controls (Vanlerberghe and Ordog, 2002; Finnegan *et al.*, 2004; Clifton *et al.*, 2006; Van Aken *et al.*, 2009; Moore *et al.*, 2013).

Important pioneering studies used AOX inhibitors such as salicylhydroxamic acid to examine how the absence of AOX activity would impact on photosynthetic performance (Raghavendra and Padmasree, 2003). In recent years, the availability of AOX knockdown/knockout plants, particularly in the model species *Arabidopsis thaliana*, have greatly aided such work. The majority of these more recent studies have used immediate and short-term (usually a few hours) increases in irradiance (high light stress) as a means to rapidly challenge chloroplast energy balance and then evaluate the importance of AOX in correcting such imbalances (Zhang *et al.*, 2010; Florez-Sarasa *et al.*, 2011; Yoshida *et al.*, 2011*a*, *b*; Vishwakarma *et al.*, 2014; Watanabe *et al.*, 2016). In general, these studies have concluded that the absence of AOX does result in some perturbation of photosynthetic metabolism following short-term increases in irradiance. One study showed that this was associated with an exaggerated increase in reduction state of the plastoquinone (PQ) and ubiquinone pools in the AOX knockdown relative to the wild-type (WT), following the shift to higher irradiance (Yoshida *et al.*, 2011*a*). Such a result suggests that AOX activity not only impacts on the mitochondrion, but on the chloroplast as well.

Other studies and types of analyses also suggest that AOX respiration has a role in optimizing leaf metabolism in response to changes in irradiance. For example, the short-term shifts to higher irradiance have been shown to rapidly increase the amount of leaf AOX transcript and/or protein in different plant species (Yoshida *et al.*, 2007, 2008, 2011*a*; Dinakar *et al.*, 2010; Wallstrӧm *et al.*, 2014). It has also been shown, in the shade species *Alocasia odora*, that upon a shift to higher irradiance, existing AOX protein is converted from an oxidized inactive to a reduced active form (Noguchi *et al.*, 2005). De-etiolation is also associated with an increase in the *AOX1a* transcript in both Arabidopsis (Rosso *et al.*, 2009; Zhang *et al.*, 2016) and wheat (Garmash *et al.*, 2015). AOX transcript amounts also display a diurnal pattern, with the highest amounts occurring early in the light period (Dutilleul *et al.*, 2003; Cvetkovska and Vanlerberghe, 2012).

Fewer studies have compared AOX transcript and/or protein amounts following long-term growth and development at different irradiances. The AOX protein amount did not differ between *Cucumis sativus* plants grown at a photosynthetic photon flux density (PPFD) of 150 and 400 µmol photons m^–2^ s^–1^ (Florez-Sarasa *et al.*, 2009) or between Arabidopsis plants grown at 80 and 350 PPFD (Yoshida *et al.*, 2011*a*). However, other studies reported that AOX transcript/protein and/or capacity were higher following growth and development at higher irradiances (Bartoli *et al.*, 2006; Florez-Sarasa *et al.*, 2011; Igamberdiev *et al.*, 2014; Shabnam *et al.*, 2015). Such findings are in keeping with analytical modelling of metabolism, which predicts the increased involvement of non-energy-conserving pathways at higher irradiances (Buckley and Adams, 2011; Cheung *et al.*, 2015). These models predict that, with increasing irradiance, increased AOX respiration is required to progressively increase the mitochondrial consumption of excess reductant while also progressively decreasing the mitochondrial contribution to ATP synthesis, being compensated instead by ATP synthesis in the chloroplast. *In planta*, the steady-state activities of AOX and cytochrome oxidase (i.e. their respective rates of oxygen consumption) can only be determined using an oxygen isotope discrimination technique (Guy *et al.*, 1989; Robinson *et al.*, 1995). However, this technique has thus far only been developed for respiration measurements in the dark, owing to the complexities of leaf oxygen exchange in the light during photosynthesis. Hence, the use of AOX transgenic/mutant plants remains an essential means to evaluate the role of AOX respiration during photosynthesis.

As outlined above, many studies have used AOX knockdown/knockout plants to evaluate whether AOX respiration is necessary to support leaf metabolism following a short-term increase in irradiance. Surprisingly, however, few studies have utilized such plants to evaluate whether higher irradiances in the long-term (i.e. higher growth irradiances) necessitate higher AOX to optimally support metabolism. To our knowledge, the only such studies are those of Yoshida and colleagues, comparing photosynthesis of wild-type and *aox1a* knockout Arabidopsis plants (Yoshida *et al.*, 2011*a*, *b*). Following long-term growth at low to medium irradiances (40 to 350 PPFD), these authors found little if any differences in photosynthesis between the WT and *aox1a*. Again, however, differences in photosynthetic performance were evident between the plant lines following short-term high light stress treatments. The Yoshida *et al.* (2011*b*) study reported no difference in the rate of respiration in the dark (*R*_D_) in the mutant. This corresponds with several other studies reporting no change (or even an increase) in *R*_D_ in this mutant compared to the WT under varied growth conditions (Giraud *et al.*, 2008; Watanabe *et al.*, 2008; Strodtkӧtter *et al.*, 2009; Florez-Sarasa *et al.*, 2011; Gandin *et al.*, 2012; Vishwakarma *et al.*, 2014; Kühn *et al.*, 2015). To our knowledge, rates of respiration in the light (*R*_L_) in this mutant have not yet been reported. In another study, it was reported that knockdown of an *AOX2* gene in soybean reduced the light-saturated rate of photosynthesis, which was attributed to a biochemical, as opposed to stomatal, limitation of photosynthesis (Chai *et al.*, 2010). However, this study did not compare plants across a range of growth irradiances. These plants exhibited no change in *R*_D_, while *R*_L_ was not examined. Hence, further studies are clearly required to establish whether AOX respiration is important only for response to short-term irradiance shifts or whether it also has an important role in the longer-term acclimation of leaf metabolism to different growth irradiances.

Both laboratory and field studies have shown that the amount of AOX protein in leaf tissue responds dynamically to seasonal and environmental parameters such as irradiance, temperature, and water availability (Taylor *et al.*, 2002; Armstrong *et al.*, 2008; Searle and Turnbull, 2011; Searle *et al.*, 2011*a*, *b*; Vanlerberghe, 2013; Dahal and Vanlerberghe, 2017). Transcriptome and other studies have shown that AOX gene(s) are amongst the most dynamic of the nuclear genes encoding mitochondrial components in response to numerous stress treatments (Clifton *et al.*, 2006). Significant progress is being made to identify the molecular components that control AOX gene expression (Ng *et al.*, 2014). However, despite these important advances, there remains limited insight into the cellular and metabolic conditions that drive the dynamic changes in AOX protein amount seen under different growth conditions.

Here, we have compared respiration and photosynthesis of WT tobacco to that of AOX knockdown and overexpression plants across a wide range of growth irradiances, and under both optimal and abiotic stress (drought) conditions. We provide evidence that AOX respiration is necessary to promote chloroplast energy balance and to optimize photosynthesis, particularly under conditions that combine high growth irradiance and abiotic stress. We also show that differences in AOX protein amount across a wide range of growth conditions correlate strongly with differences in chloroplast energy balance, suggesting that the AOX respiration capacity of leaves is tightly linked to chloroplast metabolic status.

## Materials and methods

### Plant materials and growth conditions

Tobacco (*Nicotiana tabacum* L. cv Petit Havana SR1) was used for all experiments. Transgenic plant lines with elevated levels of AOX protein (B7, B8) due to the presence of an *AOX1a* transgene driven by a constitutive 35S promoter, or suppressed levels of AOX protein (RI9, RI29) due to the presence of an *AOX1a* RNA interference construct have been described previously (Wang *et al.*, 2011, Wang and Vanlerberghe, 2013; Cvetkovska *et al.*, 2014). Seeds were germinated in vermiculite for 16 d, and seedlings were subsequently transplanted individually to 10-cm plastic pots, each containing equal amounts of a general purpose growing medium that consisted of four parts soil (Pro-mix BX, Premier Horticulture, Rivière-du-Loup, QC, Canada) and one part vermiculite. The plants were then raised in controlled-environment growth chambers (Models PGR-15 and PGC-20, Conviron, Winnipeg, MB, Canada) with a 16-h photoperiod, temperature of 28/22 ºC (light/dark), relative humidity of 60%, and photosynthetic photon flux density (PPFD) of either 150, 400, or 700 µmol photons m^–2^ s^–1^. Plants in the growth chambers were irrigated with 1/10th-strength Hoagland’s solution daily for 19 d (400 and 700 PPFD-grown plants) or 21 d (150 PPFD-grown plants), producing plants of approximately equal size across the growth irradiances. Then, water was withheld from plants for up to an additional 8 d. All analyses were performed on the fully developed 5th leaves of ‘well-watered’ plants, analyzed at 1 d following their last irrigation, or ‘drought-stressed’ plants, analyzed at 2 to 8 d following their last irrigation.

### Gas exchange and Chl *a* fluorescence

Leaf CO_2_ exchange and Chl *a* fluorescence from photosystem II (PSII) were measured simultaneously at 4 to 5 h into the light period using a portable system (GFS-3000, Heinz Walz GmbH, Effeltrich, Germany). Light was provided through red and blue LEDs (Model 3055-FL, Heinz Walz GmbH). Gas flow rate was set to 750 µmol s^–1^ and impeller (fan) speed to step 7. Photosynthetic parameters were measured at both the growth irradiance of the plant (150, 400, or 700 PPFD) and at a saturating irradiance of 1600 PPFD.

Gas exchange data were used to calculate the net CO_2_ assimilation rate (*A*) and stomatal conductance (*g*_s_) (von Caemmerer and Farquhar, 1981; Farquhar and Sharkey, 1982). Throughout this paper, respiration rate refers to rates of non-photorespiratory CO_2_ efflux. Respiration in the dark (*R*_D_) was estimated following a 30-min pre-incubation in the dark. Respiration in the light (*R*_L_) was estimated by the Kok method (Kok, 1948), as described previously (Dahal *et al.*, 2014). In brief, *A* was measured at eight irradiances between 0 and 120 PPFD, which generated a Kok break-point of approximately 20 PPFD. *R*_L_ was then estimated by extrapolating to 0 PPFD the linear relationship between *A* and PPFD over the range of 20 to 120 PPFD. Over this irradiance range, lines of best-fit had typical *r*^2^ values in the WT of 0.997 (150 PPFD-grown plants), 0.988 (400 PPFD-grown), and 0.986 (700 PPFD-grown).

Chlorphyll fluorescence analyses were done following a dark adaptation period of at least 30 min. Minimum fluorescence (*F*_o_), maximal fluorescence in the dark-adapted leaf (*F*_m_) or light-adapted leaf (*F*_m_′), steady state fluorescence in the light-adapted leaf (*F*_s_), and minimal fluorescence in the light-adapted leaf (*F*_o_′) were determined, as described previously (Maxwell and Johnson, 2000). The maximal quantum yield of PSII was calculated as: *F*_v_/*F*_m_ = (*F*_m_ – *F*_o_)/*F*_m_, while the effective quantum yield (operating efficiency) of PSII was calculated as: *Φ*_PSII_ = (*F*_m_′ – *F*_s_)/*F*_m_′ (Genty *et al.*, 1989). The rate of linear electron transport was calculated as: ETR = (*Φ*_PSII_) × (PPFD) × (0.84) × (0.5), where 0.84 and 0.5 represent estimates that leaves absorb 84% of incident photons and that 50% of these are absorbed by PSII (Yamori *et al.*, 2011). Photochemical energy quenching (qP or qL) was calculated using either the puddle model [qP = (*F*_m_′ – *F*_s_)/(*F*_m_′ – *F*_o_′)] or the lake model [qL = (*F*_m_′ – *F*_s_)/(*F*_m_′ – *F*_o_′) × *F*_o_′/*F*_s_] (Kramer *et al.*, 2004). The fraction of closed (reduced) PSII reaction centres, also known as the excitation pressure (EP), was then calculated as either 1 – qP or 1 – qL. The puddle model and the lake model generated similar results, so only EP data based on the puddle model (i.e. 1 – qP) is reported here. Non-photochemical energy quenching (NPQ), a measure of heat dissipation of absorbed light energy, was calculated as: NPQ = (*F*_m_ – *F*_m_′)/*F*_m_′ (Maxwell and Johnson, 2000).

### Protein and transcript analyses

Following respiration and photosynthesis measurements, leaves were harvested and ground to a fine powder using liquid N_2_ and a mortar and pestle. Leaf protein was then extracted as described previously (Busch *et al.*, 2007), and protein concentration of the extracts was determined by a modified Lowry method (Larson *et al.*, 1986). Immunoblot analyses were then performed as described previously (Dahal *et al.*, 2014) using primary antibodies (Agrisera, Vännäs, Sweden) raised against LHCB2 (a nuclear-encoded Chl *a*/*b* binding protein of PSII) and AOX. The signals were quantified using an image analysis system (Chemidoc XRS+ with IMAGE LAB software v.3.0; BioRad Laboratories, Mississauga, ON, Canada).

Total RNA was extracted from frozen leaf tissue using TRIzol reagent (Life Technologies) by the method described by Vanessa *et al.* (2008), and then treated with RNase-free DNase I (Life Technologies). The extracted RNA had A_260_/A_280_ and A_260_/A_230_ ratios >2. Comparative quantification of gene transcripts was performed using reverse transcription quantitative polymerase chain reaction (RT-qPCR). Gene-specific primers [*EF-1α* (AF120093), 5′-GGTACTGTCCCTGGTTGGTCG-3′, 5′-TGAAGAGCTT CGTGGTGCAT-3′; and *AOX1a* (X79768), 5′-GACAACATA CACGGAGAGTGGAGTC-3′, 5′-GTGGGTTACTTGGAAGAA GAGGC-3′] were designed using the NCBI primer design resources (www.ncbi.nlm.nih.gov). First-strand cDNA was synthesized from 1 µg of total RNA using SuperScript II RT (Life Technologies). qPCR was performed using a SYBR Green Jumpstart Taq ReadyMix (Sigma-Aldrich, Oakville, ON, Canada). Amplification (with three technical replicates) was monitored on a PTC-200 DNA Engine Thermal Cycler (Bio-Rad Laboratories) using the program: 3 min at 95 °C, followed by 39 cycles of amplification with 10 s of denaturation at 95 °C, 15 s of annealing at 59 °C and 30 s of extension at 72 °C. Amplification efficiency was 100–110% for each gene. Comparative quantification was by the ΔΔC_t_ method, with *EF-1α* as the normalizer gene.

### Other methods

Leaf water status was determined by measuring relative water content (RWC), as described previously (Wang and Vanlerberghe, 2013). Statistical analyses were conducted using PRISM 5.0 (GraphPad Software Inc., La Jolla, CA, USA).

## Results

### In well-watered tobacco, leaf AOX amount has little impact on photosynthetic and respiratory rates, regardless of growth irradiance

Tobacco plants were raised to similar size at either low (150 PPFD), medium (400 PPFD), or high (700 PPFD) irradiance. In the WT plants, the amount of leaf *AOX1a* transcript increased with growth irradiance. *AOX1a* transcript was 1.7-fold higher at 400 PPFD and 2.5-fold higher at 700 PPFD, compared with the 150 PPFD-grown plants (see Supplementary Fig. S1 at *JXB* online).

To determine if AOX was critical in supporting leaf metabolism, particularly at the higher growth irradiances, we compared respiration and photosynthesis of well-watered WT plants to that of two AOX knockdowns (RI9, RI29) and two AOX overexpressors (B7, B8), following long-term growth and development at either low, medium, or high irradiance. Table 1 summarizes the results of this comparison. In the WT plants, both respiration rates in the dark (*R*_D_) and light (*R*_L_) increased with growth irradiance. Relative to 150 PPFD-grown plants, *R*_D_ increased 1.2-fold at 400 PPFD and 1.6-fold at 700 PPFD, while *R*_L_ increased 1.6-fold at 400 PPFD and 2.5-fold at 700 PPFD. However, neither *R*_D_ nor *R*_L_ differed across the five plant lines differing in AOX protein amount, regardless of growth irradiance (Table 1).

**Table 1.** Effect of growth irradiance on respiration and photosynthesis in well-watered WT tobacco, AOX overexpressors (B8, B7) and AOX knockdowns (RI9, RI29). Data are the average ±SE of three to five independent experiments. Within an irradiance, values not sharing a common superscript letter are significantly different from one another (*P*<0.05). In data sets without superscript letters, there are no significant differences between plant lines. See text for further details.

	**Growth irradiance (µmol photons m** ^**–2**^ **s** ^**–1**^)														
	150 PPFD					400 PPFD					700 PPFD				
	B8	B7	WT	RI9	RI29	B8	B7	WT	RI9	RI29	B8	B7	WT	RI9	RI29
*F*_v_/*F*_m_	0.82 ± 0.02	0.80 ± 0.01	0.82 ± 0.01	0.81 ± 0.02	0.80 ± 0.02	0.81 ± 0.01	0.80 ± 0.02	0.79 ± 0.01	0.82 ± 0.01	0.82 ± 0.01	0.80 ± 0.02	0.79 ± 0.01	0.81 ± 0.02	0.80 ± 0.01	0.82 ± 0.02
*R*_D_ (µmol CO_2_ m^–2^ s^–1^)	0.72 ± 0.04	0.77 ± 0.02	0.83 ± 0.04	0.87 ± 0.03	0.78 ± 0.05	0.97 ± 0.06	0.93 ± 0.08	0.99 ± 0.05	1.10 ± 0.06	0.89 ± 0.08	1.25 ± 0.07	1.48 ± 0.15	1.32 ± 0.10	1.19 ± 0.09	1.35 ± 0.06
*R*_L_ (µmol CO_2_ m^–2^ s^–1^)	0.64 ± 0.04	0.61 ± 0.05	0.72 ± 0.03	0.69 ± 0.05	0.64 ± 0.05	1.05 ± 0.07	1.09 ± 0.09	1.17 ± 0.12	1.07 ± 0.09	1.16 ± 0.08	1.72 ± 0.2	1.93 ± 0.14	1.81 ± 0.16	1.67 ± 0.13	1.79 ± 0.18
**Measured at growth PPFD**															
*A*_net_ (µmol CO_2_ m^–2^ s^–1^)	3.09 ± 0.28	3.37 ± 0.47	3.49 ± 0.24	3.65 ± 0.27	3.73 ± 0.32	7.40 ± 0.56	7.35 ± 0.15	7.75 ± 0.54	7.26 ± 0.28	6.91 ± 0.72	9.63 ± 0.88	10.13 ± 0.47	10.19 ± 0.48	9.37 ± 0.37	8.71 ± 0.60
*g*_snet_ (mol CO_2_ m^–2^ s^–1^)	0.066 ± 0.003	0.062 ± 0.012	0.065 ± 0.008	0.069 ± 0.005	0.067 ± 0.004	0.087 ± 0.036	0.098 ± 0.020	0.072 ± 0.014	0.76 ± 0.05	0.068 ± 0.016	0.171 ± 0.031	0.156 ± 0.027	0.181 ± 0.16	0.149 ± 0.023	0.165 ± 0.038
*Φ*_PSIInet_	0.73 ± 0.14	0.71 ± 0.11	0.76 ± 0.08	0.74 ± 0.12	0.70 ± 0.15	0.49 ± 0.09	0.54 ± 0.06	0.51 ± 0.08	0.47 ± 0.10	0.52 ± 0.05	0.38 ± 0.04	0.34 ± 0.05	0.37 ± 0.02	0.39 ± 0.02	0.33 ± 0.04
ETR_net_ (µmol e^–^ m^–2^ s^–1^)	45 ± 1.2	47 ± 1.7	41 ± 2.29	45 ± 1.3	42 ± 2.7	80 ± 3.1	77 ± 5.3	90 ± 8.6	78 ± 5.7	82 ± 1.5	105 ± 2.4	110 ± 5.2	120 ± 9.1	116 ± 9.1	115 ± 4.4
NPQ_net_	0.19 ± 0.03	0.20 ± 0.02	0.20 ± 0.03	0.17 ± 0.02	0.18 ± 0.03	0.77 ± 0.10	0.78 ± 0.10	0.79 ± 0.11	0.76 ± 0.05	0.82 ± 0.06	1.23 ± 0.21	1.06 ± 0.17	1.20 ± 0.16	1.24 ± 0.15	1.35 ± 0.17
**Measured at 1600 PPFD**															
*A*_sat_ (µmol CO_2_ m^–2^ s^–1^)	10.72 ± 0.44^ab^	11.13 ± 0.48^ab^	11.55 ± 0.44^a^	10.71 ± 0.70^ab^	9.45 ± 0.40^b^	11.87 ± 1.04	12.42 ± 0.82	12.94 ± 0.39	10.70 ± 0.89	12.08 ± 0.42	13.17 ± 0.56^ab^	14.49 ± 0.74^a^	13.51 ± 0.41^a^	12.92 ± 0.34^ab^	11.29 ± 0.52^b^
*g*_ssat_ (mol CO_2_ m^–2^ s^–1^)	0.166 ± 0.01^ab^	0.178 ± 0.021^a^	0.183 ± 0.019^a^	0.147 ± 0.01^bc^	0.139 ± 0.006^c^	0.171 ± 0.014^b^	0.203 ± 0.003^a^	0.212 ± 0.006^a^	0.170 ± 0.004^b^	0.166 ± 0.013^b^	0.226 ± 0.007	0.218 ± 0.033	0.232 ± 0.025	0.213 ± 0.008	0.225 ± 0.016
*Φ*_PSIIsat_	0.214 ± 0.008	0.203 ± 0.005	0.219 ± 0.016	0.192 ± 0.010	0.227 ± 0.009	0.223 ± 0.021	0.235 ± 0.008	0.201 ± 0.015	0.209 ± 0.062	0.216 ± 0.053	0.202 ± 0.010	0.189 ± 0.021	0.182 ± 0.017	0.166 ± 0.051	0.179 ± 0.043
ETR_sat_ (µmol e^–^ m^–2^ s^–1^)	152 ± 10.3	147 ± 8.3	154 ± 6.2	143 ± 6.7	158 ± 4.1	143 ± 5.5	141 ± 9.8	151 ± 9.6	156 ± 7.8	148 ± 11.7	136 ± 12.9	130 ± 14.3	121 ± 0.71	136 ± 6.6	128 ± 6.7
NPQ_sat_	2.08 ± 0.15	1.89 ± 0.08	1.97 ± 0.10	1.88 ± 0.09	1.96 ± 0.08	1.77 ± 0.11	1.71 ± 0.05	1.83 ± 0.17	1.70 ± 0.09	1.72 ± 0.16	1.53 ± 0.11	1.66 ± 0.10	1.55 ± 0.08	1.61 ± 0.15	1.68 ± 0.11

In examining photosynthesis, measurements were taken at both the growth irradiance (150, 400, or 700 PPFD) and at a saturating irradiance of 1600 PPFD (Table 1). For WT plants, the CO_2_ assimilation rate (*A*) measured at growth irradiance (termed *A*_net_) increased from 3.5 µmol CO_2_ m^–2^ s^–1^ in 150 PPFD-grown plants to 7.8 µmol CO_2_ m^–2^ s^–1^ (400 PPFD-grown) and 10.2 µmol CO_2_ m^–2^ s^–1^ (700 PPFD-grown). However, no differences were observed across the five plant lines. When measured at growth irradiance, there were also no differences in stomatal conductance (*g*_snet_), photosystem II (PSII) operating efficiency (*Ф*_PSIInet_), linear electron transport rate (ETR_net_), or non-photochemical quenching (NPQ_net_) across the five plant lines, regardless of growth irradiance (Table 1).

For WT plants, the *A* measured at saturating irradiance (termed *A*_sat_) increased slightly with each increase in growth irradiance [from 11.6 µmol CO_2_ m^–2^ s^–1^ (150 PPFD-grown), to 12.9 µmol CO_2_ m^–2^ s^–1^ (400 PPFD-grown), to 13.5 µmol CO_2_ m^–2^ s^–1^ (700 PPFD-grown)]. Again, there were no differences in *A*_sat_ across the plant lines, except for a slightly lower rate in one of the knockdown lines grown at 150 or 700 PPFD (Table 1). This probably relates to our previous report that knockdown of AOX can impact on stomatal function when photosynthesis of well-watered plants is measured at saturating irradiance (Cvetkovska *et al.*, 2014). In keeping with this, *g*_ssat_ also differed slightly in both knockdowns for plants grown at 150 or 400 PPFD. Nonetheless, there were no differences in other key photosynthetic parameters (*Ф*_PSIIsat_, ETR_sat_, NPQ_sat_) across the plant lines, regardless of growth irradiance. In summary, AOX amount (knockdown or overexpression) was found to have little if any impact on photosynthetic performance across a wide range of growth and measurement irradiances in well-watered plants (Table 1).

### In drought-stressed tobacco, AOX amount is an important determinant of photosynthetic and respiratory rates, particularly during growth at higher irradiances

We previously established that tobacco AOX is critical to maintain respiration and photosynthesis during drought (Dahal *et al.*, 2014, 2015). These previous studies were done at a low growth irradiance of 150 PPFD. To further evaluate whether AOX respiration takes on added importance at higher irradiances, plants grown at different irradiances (150, 400, and 700 PPFD) were subjected to drought. Our hypothesis was that the importance of AOX in maintaining respiration and photosynthesis during drought would be exaggerated at higher growth irradiances.

**Fig. 1.**
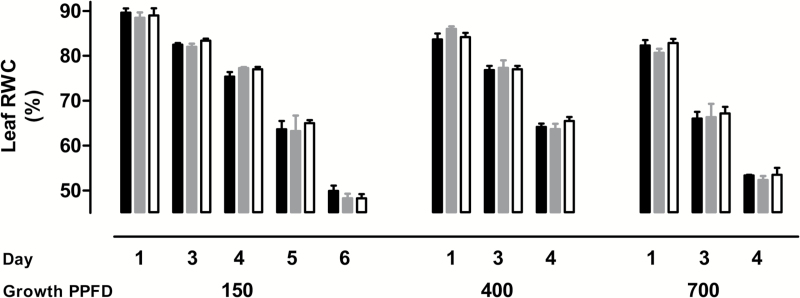
Leaf RWC at different growth irradiances (150, 400, or 700 PPFD) and at different times following the withholding of water. ‘Day’ represents the number of days since water was withheld from well-watered plants. Data are shown for WT plants (gray bars), AOX overexpressors (black bars), and AOX knockdowns (white bars). In each independent experiment, data from two overexpressors (B7 and B8) that acted similarly were averaged, and data from two knockdowns (RI9 and RI29) that acted similarly were averaged. The data shown are the average ±SE of three to five independent experiments.

On Day 0, water was withheld from plants growing at 150, 400, or 700 PPFD. Plant water status was then assessed over time by examining leaf relative water content (RWC) (Fig. 1). Day 1 plants (i.e. one day following their last watering) were defined as well-watered, when WT plants had a RWC of approximately 89% (150 PPFD-grown), 86% (400 PPFD-grown), or 81% (700 PPFD-grown). Further declines in RWC over time occurred more rapidly at higher growth irradiance. For example, on Day 4, the RWC of the WT leaf was 77% in 150 PPFD-grown plants, 64% in 400 PPFD-grown plants, and only 52% in 700 PPFD-grown plants (Fig. 1). AOX amount had no influence on RWC. On all days and at all growth irradiances, there were no significant differences in leaf RWC between the five plant lines (Fig. 1). Hence, differences in respiration and photosynthesis between plant lines (see below) cannot be attributed to differences in leaf water status between lines.

**Fig. 2.**
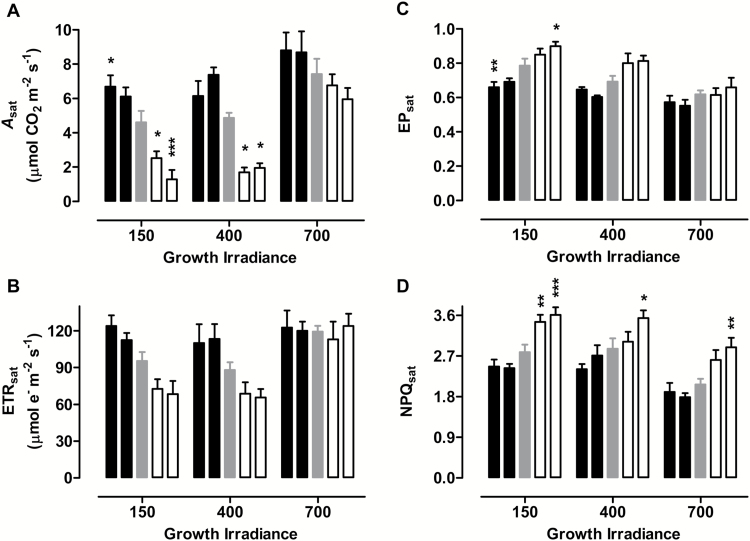
Effect of growth irradiance on photosynthesis during moderate drought in WT tobacco and transgenic lines with altered amounts of AOX protein. (A) *A*_sat_. (B) ETR_sat_. (C) EP_sat_. (D) NPQ_sat_. Plants were grown at 150, 400, or 700 PPFD under well-watered conditions for 19 to 21 days, followed by water being withheld from the plants until they were experiencing a leaf RWC of 63–66%, at which time each of the photosynthetic parameters was measured at saturating irradiance (1600 PPFD). Data are shown for WT plants (gray bars), AOX overexpressors (left to right the two black bars are B8 and B7, respectively), and AOX knockdowns (left to right the two white bars are RI9 and RI29, respectively). Data are the average ±SE of three to five independent experiments. Within an irradiance, values significantly different from the WT are indicated: *P<0.05; **P<0.01; ***P<0.001.

The response of photosynthesis and respiration to increasing leaf water deficit was compared across the plant lines and at each of the three growth irradiances. Since the water status of plants at different irradiances declined at different rates (as noted above), these comparisons were done as a function of leaf RWC. At high RWC (>80%), there was little if any difference in *A*_sat_, ETR_sat_, EP_sat_ [the proportion of closed (reduced) PSII reaction centres, known as excitation pressure (EP) and measured as the fluorescence parameter 1 – qP)], or NPQ_sat_ across the plant lines, regardless of growth irradiance (see Supplementary Fig. S2). However, with further declines in RWC, differences emerged between lines. For 150 PPFD-grown plants, *A*_sat_ and ETR_sat_ were enhanced in AOX overexpressors and reduced in AOX knockdowns relative to the WT, while EP_sat_ and NPQ_sat_ were enhanced in knockdowns and reduced in overexpressors relative to WT (Supplementary Fig. S2A–D). This corresponds with our previous work at 150 PPFD (Dahal *et al.*, 2014, 2015). A similar pattern across plant lines was also evident in the 400 PPFD- and 700 PPFD-grown plants experiencing low RWC’s and measured at saturating irradiance (Supplementary Fig. S2E–L). Importantly, the magnitude of the differences between plant lines was similar, regardless of whether the plants were experiencing the low RWC at low, medium, or high growth irradiance. This conclusion is most easily illustrated and summarized by Fig. 2, in which plants at different growth irradiances are compared at the similar, and moderately low, RWC of 63% to 66%. This analysis shows that the differences in photosynthetic parameters between plant lines grown at 150 PPFD were not exaggerated at the higher growth irradiances. In fact, 700-PPFD grown plants showed the least differences in photosynthetic parameters across plant lines (Fig. 2).

**Fig. 3.**
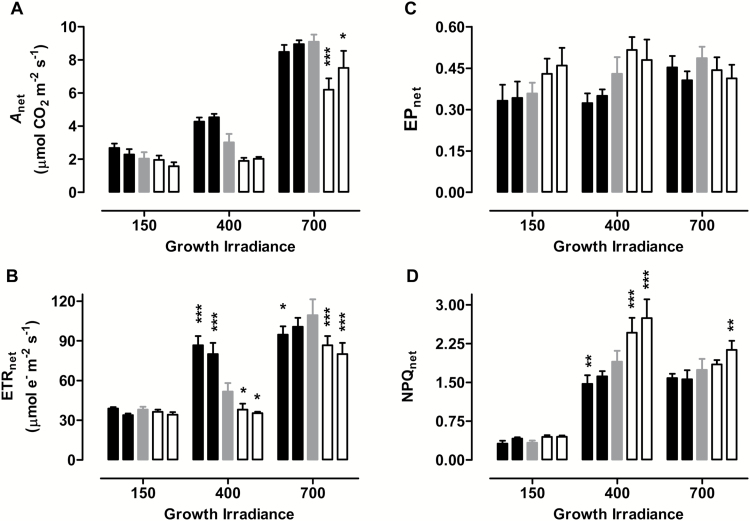
Effect of growth irradiance on photosynthesis during moderate drought in WT tobacco and transgenic lines with altered amounts of AOX protein. (A) *A*_net_. (B) ETR_net_. (C) EP_net_. (D) NPQ_net_. Plants were grown at 150, 400, or 700 PPFD under well-watered conditions for 19 to 21 days, followed by water being withheld from the plants until they were experiencing a leaf RWC of 63–66%, at which time each of the photosynthetic parameters was measured at the growth irradiance (150, 400, or 700 PPFD). Data are shown for WT plants (gray bars), AOX overexpressors (left to right the two black bars are B8 and B7, respectively) and AOX knockdowns (left to right the two white bars are RI9 and RI29, respectively). Data are the average ±SE of three to five independent experiments. Within an irradiance, values significantly different from the WT are indicated: *P<0.05; **P<0.01; ***P<0.001.

The above analysis was repeated, but with measurements done at the growth irradiance rather than a saturating irradiance. Now, for 150 PPFD-grown plants there were only modest differences in photosynthetic parameters between the plant lines at lower RWCs (see Supplementary Fig. S3A–D). Under these measurement conditions, the higher growth irradiances (400 and 700 PPFD) combined with low RWC resulted in greater differences in photosynthesis between the plant lines (Supplementary Fig. S3E–L). Again, this conclusion is most easily illustrated and summarized by Fig. 3, in which plants at different growth irradiances are compared at the similar, and moderately low, RWC of 63 to 66%. This analysis shows that the differences in photosynthetic parameters between plant lines grown at 150 PPFD and experiencing moderate drought are exaggerated at higher growth irradiances. This is particularly the case for *A*_net_, ETR_net_, and NPQ_net_ (Fig. 3).

**Fig. 4.**
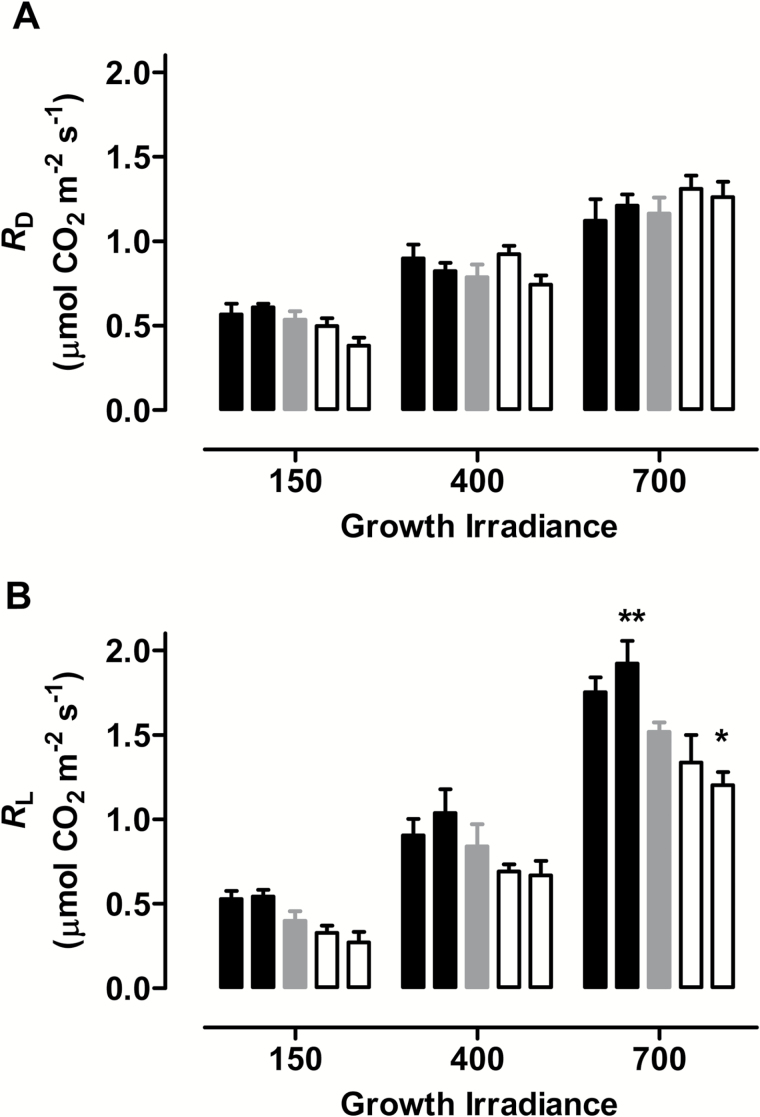
Effect of growth irradiance on respiration during moderate drought in WT tobacco and transgenic lines with altered amounts of AOX protein. (A) *R*_D_. (B) *R*_L_. Plants were grown at 150, 400, or 700 PPFD under well-watered conditions for 19 to 21 days, followed by water being withheld from the plants until they were experiencing a leaf RWC of 63–66%, at which time the measurements were taken. Data are shown for WT plants (gray bars), AOX overexpressors (left to right the two black bars are B8 and B7, respectively) and AOX knockdowns (left to right the two white bars are RI9 and RI29, respectively). Data are the average ±SE of three to five independent experiments. Within an irradiance, values significantly different from the WT are indicated: *P<0.05; **P<0.01; ***P<0.001.

At high RWC (>80%), there was little difference in *R*_D_ or *R*_L_ across the plant lines, regardless of growth irradiance (see Supplementary Fig. S4 at *JXB* online). With further declines in RWC, a modest decrease in *R*_D_ occurred similarly across the plant lines, regardless of growth irradiance. *R*_L_ also declined with RWC but this response differed across plant lines and growth irradiances. Particularly at the highest growth irradiance, the decline in *R*_L_ was greatest in the AOX knockdown lines and least in the AOX overexpression lines. Again, these conclusions are most easily illustrated and summarized by Fig. 4, in which plants at different growth irradiances are compared at the similar, and moderately low, RWC of 63 to 66%. This analysis shows that there are no differences in *R*_D_ across plant lines (Fig. 4A) but that there are differences in *R*_L_ across lines at the higher growth irradiances, particularly 700 PPFD (Fig. 4B). Overall, these results indicate that, under drought, the amount of AOX preferentially impacted the level of *R*_L_ (over that of *R*_D_), with this effect being most pronounced at higher growth irradiances (Fig. 4). Knockdown of AOX compromised *R*_L_ relative to the WT, while overexpression enhanced *R*_L_ relative to the WT (Fig. 4B).

We also examined the ratio of *R*_L_ to *R*_D_ across the plant lines and growth conditions. In both well-watered plants (see Supplementary Fig. S5A) and drought-stressed plants (Fig. S5B), growth at higher irradiance increased the *R*_L_/*R*_D_ ratio. In well-watered plants, this ratio was similar across plant lines, regardless of growth irradiance. However, in plants experiencing moderate drought (63 to 66% RWC) at high growth irradiance (400 and 700 PPFD), the *R*_L_/*R*_D_ ratio tended to be highest in the AOX overexpressors and lowest in the knockdowns, with WT plants showing an intermediate response. These differences were not statistically significant but do suggest that AOX amount is an important determinant of the *R*_L_/*R*_D_ ratio in plants grown at high-irradiance and experiencing moderate drought.

### In the tobacco leaf, differences in AOX protein amount across growth conditions correlates strongly with differences in the reduction state of the photosynthetic electron transport chain

**Fig. 5.**
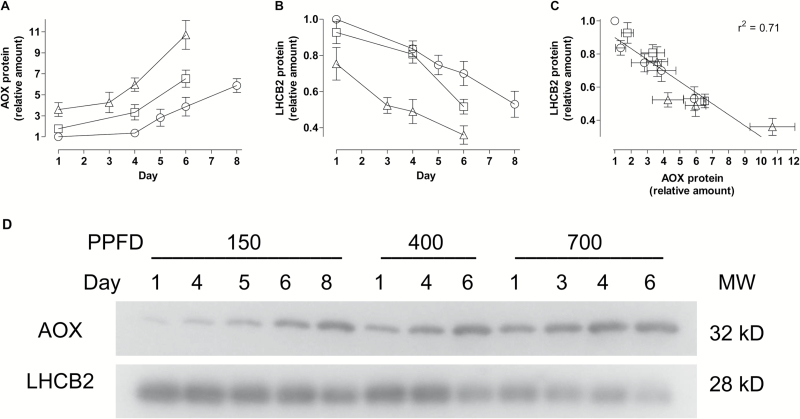
Leaf protein amounts. (A) AOX protein amount. (B) LHCB2 protein amount. (C) Plot of LHCB2 protein amount versus AOX protein amount. (D) Representative immunoblots. WT tobacco plants were grown at an irradiance of 150 PPFD (circles), 400 PPFD (squares), or 700 PPFD (triangles) under well-watered conditions for 19 to 21 days. AOX and LHCB2 protein amounts were subsequently determined at different times for up to 8 days following a final watering of the plants on Day 0. Protein amounts in (A) and (B) are relative to that of the well-watered (Day 1) plants grown at 150 PPFD, which was set to 1. Data are the average ±SE of three independent experiments. Data in (C) are derived from that in (A) and (B).

Using WT tobacco, we examined more closely the relationship between leaf AOX protein amount and growth irradiance. This included comparison with a light-harvesting complex II protein (LHCB2) whose abundance is known to respond dynamically to irradiance (Bailey *et al.*, 2001). In well-watered plants (Day 1), the AOX protein amount increased with increases in growth irradiance (Fig. 5A), while the LHCB2 protein amount declined with increases in growth irradiance (Fig 5B). Compared to 150 PPFD-grown plants, 400 PPFD- and 700 PPFD-grown plants had 1.8-fold and 3.6-fold higher AOX protein amount, respectively. On the other hand, 400 PPFD- and 700 PPFD-grown plants had only 93% and 75%, respectively, of the LHCB2 protein amount present in 150 PPFD-grown plants. The opposite response of these two proteins was also evident following the imposition of drought. In response to increasing drought severity (Days 1–8), the AOX protein amount increased while the LHCB2 protein amount declined, with these trends seen at all three growth irradiances (Fig. 5A, B). A plot of LHCB2 protein amount versus AOX protein amount shows a strong inverse relationship between these two proteins across this wide range of growth irradiances and leaf water status (Fig. 5C).

**Fig. 6.**
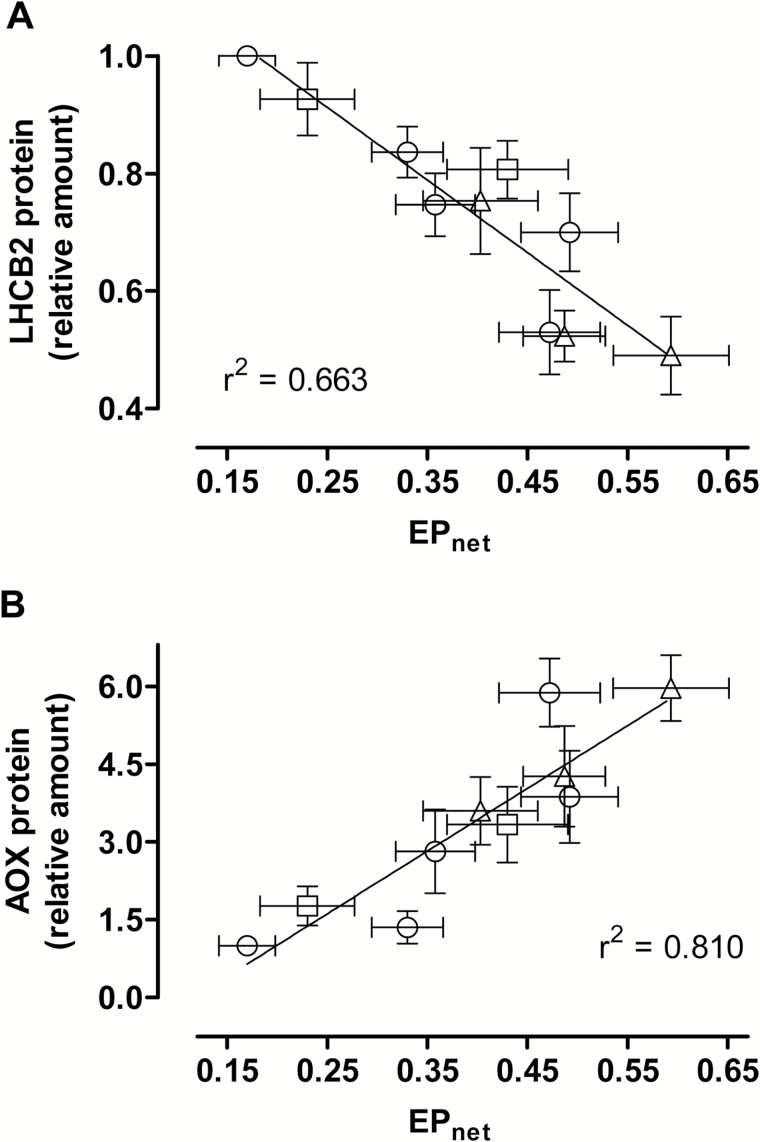
Leaf AOX and LHCB2 protein amounts and their relation to EP_net_ in WT tobacco. (A) The negative relationship between LHCB2 protein amount and EP_net_. (B) The positive relationship between AOX protein amount and EP_net_. Plants were grown at 150 PPFD (circles), 400 PPFD (squares), or 700 PPFD (triangles) under well-watered conditions for 19 to 21 days, followed by water being withheld from the plants for up to an additional 8 days. At different times following the water being withheld, AOX and LHCB2 protein amounts, as well as the EP_net_ being experienced by the plant were determined. All protein amounts are relative to that of well-watered plants grown at 150 PPFD, which was set to 1. All data are the average ±SE of three independent experiments.

Current models suggest that *LHCB2* gene expression and protein amount is responsive to signal(s) reflecting the chloroplast energy balance (Dall’Osto *et al.*, 2015; Wobbe *et al.*, 2016). One such indicator of energy balance is EP. High EP (i.e. a high proportion of closed PSII reaction centres) indicates an imbalance between light energy absorption and the downstream processes that utilize or dissipate that energy. High EP will also manifest itself as an increase in the reduction state of the PQ pool, since plastoquinol oxidation is typically the rate-limiting step in the photosynthetic electron transport chain (Huner *et al.*, 1998; Rosso *et al.*, 2009; Wobbe *et al.*, 2016). We again grew plants at the three different growth irradiances and measured the steady-state growth EP (EP_net_) being experienced by them under both well-watered conditions and in response to different severities of drought (leaf RWCs ranging from 89% to 40%). These same plants were then used to examine both LHCB2 and AOX protein amount. This analysis revealed that LHCB2 protein amount showed a strong negative relationship with EP_net_ (Fig. 6A) while AOX protein amount showed a strong positive relationship with EP_net_ (Fig. 6B) across this wide range of growth irradiances and leaf water status.

### Transgenic plants with altered AOX amount display aberrant levels of *R*_L_ and an altered reduction state of the photosynthetic electron transport chain

**Fig. 7.**
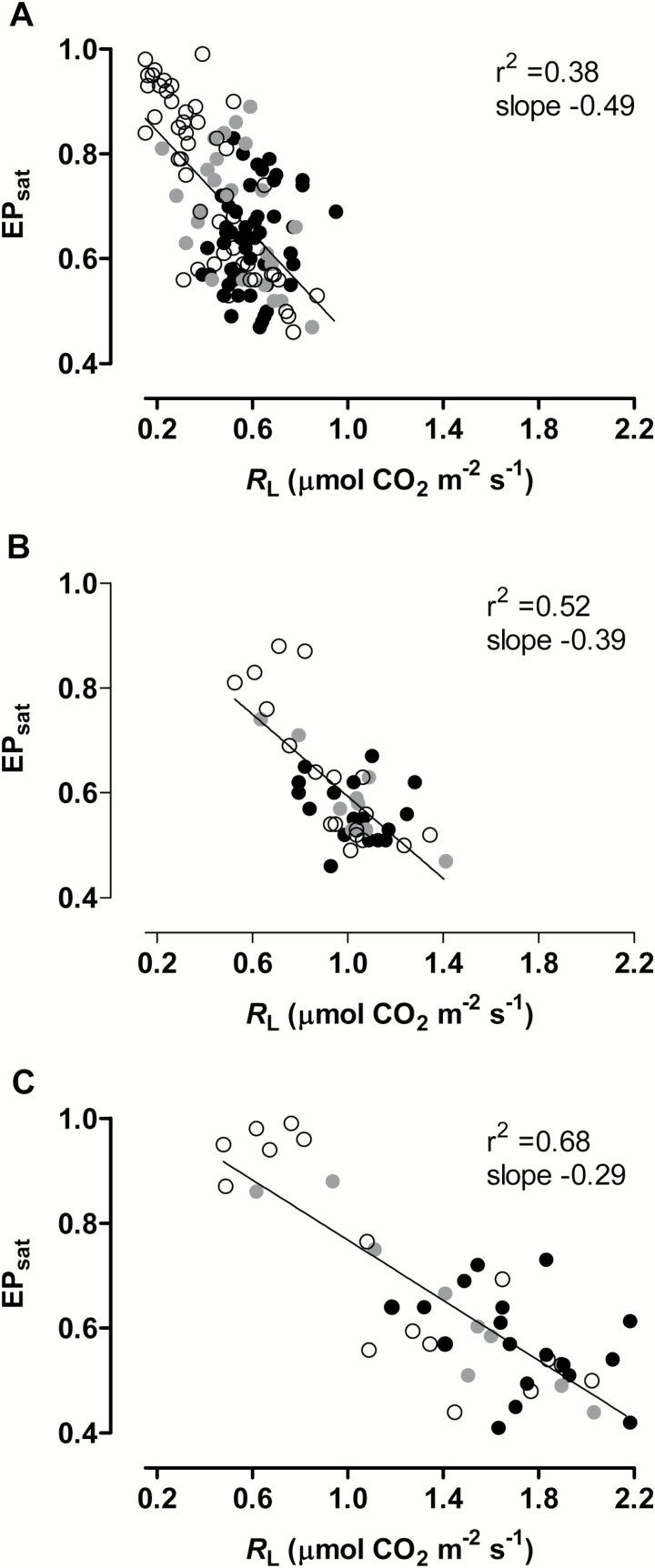
Changes in *R*_L_ and EP_sat_ as a function of growth irradiance and leaf water status in WT tobacco and transgenic tobacco lines with altered amounts of AOX protein. Plants were grown at 150 PPFD (A), 400 PPFD (B), or 700 PPFD (C) under well-watered conditions for 19 to 21 days, followed by water being withheld from the plants for up to an additional 6 days. At different times following the water being withheld, leaf *R*_L_ and EP_sat_ were determined. Data are shown for WT plants (gray circles), AOX overexpressors (black circles; half are B7, half are B8) and AOX knockdowns (white circles; half are RI9, half are RI29). The data points are compiled from three to five independent experiments.

In WT tobacco, the AOX protein amount correlated strongly with EP_net_ (Fig. 6B), suggesting that AOX (like LHCB2) may be an important player in the control of chloroplast energy balance. To test this hypothesis, we used the WT, AOX knockdown, and AOX overexpression plants to examine the interplay between *R*_L_ and chloroplast energy balance at saturating irradiance, using plants grown under the same wide range of growth irradiances and water availabilities as before (see above). In well-watered plants, EP_sat_ varied over only a relatively narrow range (of about 0.41 to 0.62), regardless of growth irradiance or plant line (see Supplementary Fig. S6). As seen before (Table 1), *R*_L_ was responsive to growth irradiance, with higher growth irradiances resulting in higher *R*_L_, but the AOX protein amount across the plant lines apparently had little influence over *R*_L_ (Supplementary Fig. S6, Table 1). Hence, in well-watered plants, the amount of AOX and *R*_L_ had little apparent influence over the chloroplast energy balance (EP_sat_), even at high growth and measurement irradiances. However, the results were quite different when data from drought-stressed plants were included in this analysis (Fig. 7). Now, the range of values measured was much greater for both EP_sat_ and *R*_L_ and, within each growth irradiance, these parameters were inversely correlated with one another. Further, the knockdown lines were responsible for most of the lowest *R*_L_ and hence highest EP_sat_ values, while the overexpression lines were responsible for most of the highest *R*_L_ and hence lowest EP_sat_ values. These data indicate that AOX amount is an important determinant of *R*_L_ during drought, which in turn is an important determinant of chloroplast energy balance. We have previously established this relationship for low-irradiance-grown plants (150 PPFD) during drought (Dahal and Vanlerberghe, 2017) and the current work indicates that this relationship during drought can be seen in plants grown at a wide range of irradiances (150, 400, and 700 PPFD). However, combining all of the data across both growth irradiance and drought severity (i.e. combining all of the data in Fig. 7A–C) does not generate a strong correlation between *R*_L_ and EP_sat_. This is because the quantitative relationship between these parameters (i.e. the value of *R*_L_ necessary to maintain any given EP_sat_) clearly differs between growth irradiances (i.e. the slopes of the line of best fit differ between Fig. 7A, B and C).

## Discussion

We compared WT tobacco plants to AOX knockdown and overexpression plants to examine whether AOX is critical in maintaining leaf respiration and photosynthesis. This comparison was done for plants grown at low, medium, and high irradiance since it has been observed in tobacco (this study) and other species (see Introduction) that plants maintain higher amounts of AOX protein at higher growth irradiance. Under optimal growth conditions (well-watered, ample nutrients), there were no differences in respiration rate (*R*_D_ or *R*_L_) or photosynthetic performance (measured at either the growth or a saturating irradiance) across the tobacco lines differing in AOX amount, regardless of whether the plants were grown at low, medium, or high irradiance (Table 1). Respiration rates (both *R*_D_ and *R*_L_) did increase with growth irradiance, as has been seen in many other studies (e.g. Bartoli *et al.*, 2006; Wright *et al.*, 2006). Nonetheless, AOX respiration was not critical in maintaining leaf metabolism, even at the highest growth irradiance.

To our knowledge, in only one other plant species has the energy metabolism of AOX knockdown and/or overexpression plants following growth and development at different irradiances been examined. Yoshida *et al.* (2011*a*, *b*) compared WT Arabidopsis to an *aox1a* knockout following growth at low to medium irradiances (40 to 350 PPFD) and, similar to the current study, found little if any impact of AOX amount on photosynthetic performance. So why do tobacco and other species maintain higher AOX protein amounts at higher growth irradiances if it is not necessary to maintain respiration and photosynthesis? One possibility is that AOX does in fact make a greater contribution to respiration at higher growth irradiances but that, under the conditions examined, the cytochrome pathway was able to compensate for the altered AOX amount in the transgenics, resulting in little overall change in respiration rate, and little consequence for photosynthesis. For example, the amount of photoinhibition resulting from a shift to high irradiance was greater in the Arabidopsis *aox1a* mutant compared to the WT, but only under conditions in which the capacity of the cytochrome pathway was being simultaneously reduced using antimycin A (Watanabe *et al.*, 2016). These results indicate that a functional mitochondrial electron transport chain (ETC) is necessary to prevent photoinhibition but do not clarify the specific importance of AOX respiration in this process. Another study compared photoinhibition across five plant species given a 2-h high irradiance treatment and found no correlation between the amount of photoinhibition and the activity of AOX during a post-treatment dark period, measured using the isotope discrimination technique (Florez-Sarasa *et al.*, 2016). It is also possible that the higher AOX protein amount seen at higher irradiances in the current study is required to support metabolism that is unrelated to photosynthesis. Another possibility relates to a hypothesis put forward by Rasmusson and co-workers who suggested that the amount of AOX protein must be sufficient to support its ‘peak activity’, which might be significantly higher than its usual activity (Rasmusson *et al.*, 2009). For example, it has been suggested that high AOX activity may be necessary during the photosynthetic induction period following darkness (Gardestrӧm and Igamberdiev, 2016). An energy imbalance could develop during this period since the energy-generating thylakoid reactions of photosynthesis become engaged more quickly that the energy-consuming Calvin cycle reactions. The isotope discrimination technique has shown that AOX usually operates at less than its maximal capacity (at least in the dark), suggesting that AOX amount is poised to allow a rapid increase in its activity if required (Millar *et al.*, 1998; Guy and Vanlerberghe, 2005; Ribas-Carbo *et al.*, 2005; Gomez-Casanovas *et al.*, 2007; Armstrong *et al.*, 2008).

Drought stress limits Calvin cycle activity since stomatal closure, intended to reduce leaf water loss, also restricts CO_2_ diffusion into the leaf (Flexas *et al.*, 2004; Pinheiro and Chaves, 2011). Under such conditions, rates of ATP and NADPH generation by the photosynthetic ETC may outpace their rates of consumption by the Calvin cycle, potentially resulting in an energy imbalance in the chloroplast. It has been shown in several species that drought can also result in a biochemical limitation of photosynthesis that reduces *A* to rates below those simply due to restricted CO_2_ availability. This biochemical limitation involves the loss of key photosynthetic components, in particular chloroplast ATP synthase (Tezara *et al.*, 1999; Kohzuma *et al.*, 2009; Hoshiyasu *et al.*, 2013). The specific metabolic conditions and signaling cascades that are responsible for these losses remain largely unknown but energy imbalances in the chloroplast have been suggested to be an important underlying factor (Lawlor and Tezara, 2009; Schӧttler and Tόth, 2014). Previously, we established that WT tobacco plants began to experience this biochemical limitation at a leaf RWC of about 65%. However, in AOX knockdown plants a lower stress severity threshold (RWC of about 75–80%) already induced this limitation, while in AOX overexpression plants a much higher stress threshold (RWC below about 55%) was necessary to induce this limitation. These results are consistent with a model whereby the non-energy-conserving nature of AOX respiration can act to alleviate chloroplast energy imbalances, hence increasing the threshold level of drought stress necessary to induce the biochemical limitations of photosynthesis (Dahal *et al.*, 2014, 2015; Dahal and Vanlerberghe, 2017).

In the current study, we found no evidence that AOX is critical in maintaining leaf metabolism at higher growth irradiances, despite the higher amounts of AOX transcript and protein in such plants. However, higher growth irradiances might exacerbate drought-induced chloroplast energy imbalances. We therefore evaluated whether the role of AOX in maintaining photosynthetic performance during drought was exaggerated at higher growth irradiances. Interestingly, the results of this analysis were dependent upon whether photosynthetic performance was evaluated at a saturating irradiance (1600 PPFD) or at the growth irradiance. Measured at our saturating irradiance, the magnitude of the differences in photosynthetic performance across the plant lines under moderate drought (63–66% RWC) was similar regardless of growth irradiance. If anything, the differences across plant lines tended to be slightly greater for plants grown at the lowest irradiance (150 PPFD). However, these results are difficult to interpret for the following reason. Plants acclimated to growth at 700 PPFD are likely to be inherently better equipped to maintain photosynthetic performance at saturating irradiance (1600 PPFD) than plants acclimated to 150 PPFD, irrespective of AOX amount, due to a wide range of acclimation processes (Schӧttler and Tόth, 2014; Tikkanen and Aro, 2014; Dietz, 2015; Wobbe *et al.*, 2016). As one indication of this, well-watered WT plants grown at 700 PPFD maintain a lower EP_sat_ and NPQ_sat_ than those grown at 150 PPFD. Hence, at the saturating irradiance we used, any defects in photosynthetic performance under drought and owing to lack of AOX may be offset, in the higher-irradiance-grown plants, by an inherent capacity to better maintain energy balance and photosynthetic performance at 1600 PPFD.

For the reasons discussed above, a less ambiguous means to evaluate the importance of AOX under drought across growth irradiances is to evaluate photosynthetic performance at the growth irradiance. When photosynthesis was evaluated at the growth irradiance, then little if any difference was seen in photosynthetic performance across the plant lines grown at 150 PPFD and experiencing moderate drought. In the higher irradiance-grown plants, however, there were now clear differences in photosynthetic performance across the plant lines when experiencing moderate drought. Importantly, these results were mirrored by differences in *R*_L_. The differences in *R*_L_ across plant lines during drought were most evident in the plants grown at the higher irradiances. Overall, our conclusion is that the importance of AOX in maintaining respiration and photosynthesis during drought is amplified at higher growth irradiance. This probably relates to the overall much higher values of *R*_L_ seen in plants grown at higher irradiances (see below).

**Fig. 8.**
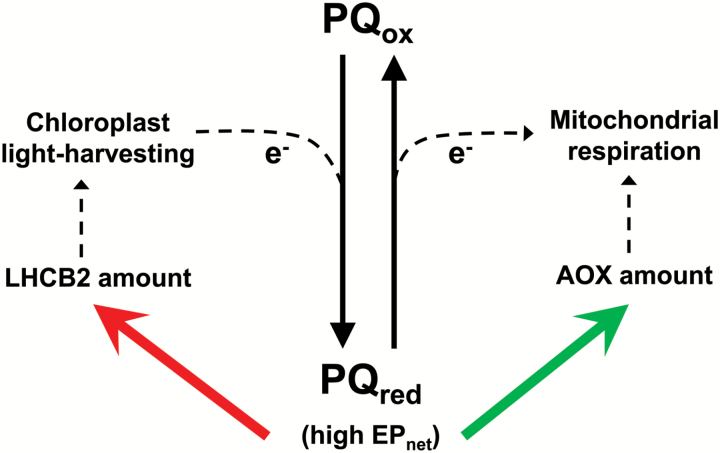
A working model for the coordinated regulation of chloroplast and mitochondrial proteins that contribute toward chloroplast energy balance. In response to growth conditions that promote a high steady-state growth excitation pressure (EP_net_), plants decrease (red arrow) chloroplast components such as LHCB2 that promote energy absorption, and increase (green arrow) mitochondrial components such as AOX that promote energy dissipation. These coordinated adjustments promote energy balance in the chloroplast by reducing electron flow into the PQ pool and increasing electron flow out of the PQ pool. This figure is highly simplified to highlight just those proteins quantified in this study (LHCB2, AOX). Many other processes also contribute toward chloroplast energy balance in response to growth conditions. PQ_ox_, oxidized plastoquinone pool; PQ_red_, reduced plastoquinone pool.

Remarkably, across a wide range of growth conditions resulting from differences in both growth irradiance and drought severity, there was a strong negative correlation in WT leaf tissue between LHCB2 and AOX protein amounts, suggesting that the abundance of these chloroplast and mitochondrial-localized components was being controlled in a coordinated and opposing manner. The abundance of many photosynthetic components is known to be responsive to changes in chloroplast energy balance (Bräutigam *et al.*, 2009; Dall’Osto *et al.*, 2015; Wobbe *et al.*, 2016). Indeed, we found a strong negative correlation between the steady-state reduction state of the PQ pool, as measured by EP_net_, and the LHCB2 protein amount. This suggests that acclimation to a high PQ-pool reduction state includes lowering the LHCB2 protein amount, presumably to lower the rates of light energy absorption that contribute to over-reduction (Fig. 8). A decline in LHCB2 at higher growth irradiance has also been reported in Arabidopsis (Bailey *et al.*, 2001). On the other hand, the AOX protein amount correlated positively with EP_net_. That is, the leaf acclimated to a high reduction state of the PQ pool by increasing the AOX protein amount (Fig. 8). The clearly opposing manner in which LHCB2 and AOX protein amounts responded to the redox status of the chloroplast ETC is precisely what might be expected if one of these components was contributing to the reduction of the PQ pool, as LHCB2 clearly does, while the other was contributing to the oxidation of the PQ pool, as we suggest to be an important role for AOX (see more below) (Fig. 8).

Based on the above finding, we suggest that signal(s) deriving from the photosynthetic ETC status may play a role in controlling leaf AOX protein abundance, similar to models in which the abundance of photosynthetic components, such as light-harvesting components, are hypothesized to be controlled (Bräutigam *et al.*, 2009; Dall’Osto *et al.*, 2015; Wobbe *et al.*, 2016). Interestingly, the transcription factor ABI4 has been identified as a regulator of AOX expression (Giraud *et al.*, 2009), but is also well recognized to control the expression of photosynthetic components through chloroplast retrograde pathways (Leόn *et al.*, 2013). Speculatively, ABI4 could represent a common molecular link allowing both AOX and LHCB2 amounts to be coordinately controlled by signals deriving from the photosynthetic ETC status.

If changes in the AOX protein amount are indeed an important acclimation supporting chloroplast energy balance, then the AOX knockdowns and overexpressors might be expected to display aberrant EPs, particularly when measured at saturating irradiance (i.e. EP_sat_). Within each growth irradiance, there was a strong negative correlation across plant lines and drought severity treatments between the level of *R*_L_ and EP_sat_. Further, AOX knockdowns tended to maintain higher EP_sat_ and lower *R*_L_ than the WT, while overexpressors tended to maintain lower EP_sat_ and higher *R*_L_ than the WT. These data clearly show the importance of AOX in lowering the reduction state of the chloroplast ETC by influencing the level of *R*_L_. A recent study reported increases in the ratio of *R*_L_/*R*_D_ in *Quercus ilex* in response to long-term drought in the field, further supporting the apparent importance of *R*_L_ during this abiotic stress (Sperlich *et al.*, 2016). On the other hand, other greenhouse and field studies have reported decreases in the *R*_L_/*R*_D_ ratio in response to drought (Ayub *et al.*, 2011; Crous *et al.*, 2012). We also saw a small decrease in *R*_L_/*R*_D_ ratio in WT tobacco in response to drought, regardless of growth irradiance. Since specific levels of *R*_L_ and *R*_D_ appear to be dependent upon many factors, including species, tissue, age, and environment (Atkin *et al.*, 2000; Wright *et al.*, 2006; Tcherkez *et al.*, 2008), we are cautious about making any broad or explicit conclusions about changes in the *R*_L_/*R*_D_ ratio.

Within each growth irradiance, *R*_L_ and EP_sat_ were tightly correlated across a wide range of drought severities. However, the quantitative relationship between EP_sat_ and *R*_L_ differed across the growth irradiances, such that at higher irradiance the control of chloroplast energy balance during drought required concomitantly higher *R*_L_. We interpret this as an example of long-term coarse control versus short-term fine control of chloroplast energy balance by respiration. In the long-term, growth at higher irradiance has been shown to increase the capacity of respiration through changes in gene expression and protein amount (Noguchi *et al.*, 1996, 2001, 2005). This allows for the higher respiratory activity typical of growth at higher irradiances (Bartoli *et al.*, 2006; Wright *et al.*, 2006). This higher respiratory activity increases the provision of carbon skeletons and energy intermediates that are necessary to support the higher growth rates typical of these conditions (Noguchi, 2005). Tobacco clearly respired faster at higher growth irradiances, particularly in the light, but also in the dark. Hence, the long-term coarse control of respiratory activity across growth irradiances fundamentally changed the quantitative relationship between *R*_L_ and the chloroplast energy balance (EP_sat_). Such coarse control is probably a primary explanation for the higher AOX transcript and protein amounts seen in tobacco at higher growth irradiance, although we found no evidence that this additional AOX was an *a priori* requirement to support respiration under these conditions. On the other hand, the relatively shorter-term (several days) progressive increase in drought stress did not alter, within a growth irradiance, the quantitative relationship between *R*_L_ and chloroplast energy balance. We view this as a short-term fine control, where a progressively increasing severity of stress and hence energy imbalance in the chloroplast is being offset by instantaneous higher *R*_L_, which is clearly AOX-dependent. This short-term fine control probably involves the activation of available AOX protein through established biochemical controls (Vanlerberghe *et al.*, 1995). However, this shorter-term control is also associated over time with increased AOX protein and, interestingly, with declines in maximal cytochrome oxidase activity (Dahal and Vanlerberghe, 2017). Hence, the fine control in response to stress is primarily acting to rebalance the partitioning of electrons between the two respiratory branches. This rebalancing, in turn, acts to ease energy imbalance in the chloroplast. This is because, at any given rate of ATP turnover, AOX respiration consumes more electrons than the cytochrome pathway. Depending upon the actual rate of mitochondrial electron transport, AOX could be used to increase the rate of pyridine nucleotide turnover and/or to reduce the rate of ATP generation, depending upon what scenario might best correct the chloroplast energy imbalance. An additional potential consequence of AOX activity in the light (though not yet investigated) is that by influencing the mitochondrial redox state, it may influence the mix of complete versus partial tricarboxylic acid (TCA) cycle activity in the light. If lower than normal AOX activity in the light increases the matrix NAD(P)H/NADP^+^ ratio, this could favor partial TCA cycle activity (see models outlined in Hurry *et al.*, 2005) and hence a reduction of TCA cycle CO_2_ efflux (i.e. lower *R*_L_). Conversely, if higher than normal AOX activity in the light lowers the matrix NAD(P)H/NAD(P)^+^ ratio, this could favor complete TCA cycle activity, hence increasing TCA cycle CO_2_ efflux (i.e. higher *R*_L_).

In summary, while growth irradiance acted primarily as a strong determinant of overall respiratory capacity and activity in both light and dark, drought stress acted primarily as a strong determinant of the partitioning of electrons between the cytochrome pathway and AOX, especially in the light. Further, while higher growth irradiances do result in higher AOX protein amounts in tobacco, as in other species, we found no evidence that this additional AOX is necessary to maintain photosynthetic performance under optimal growth conditions. On the other hand, with the imposition of drought stress this additional AOX was beneficial in optimizing photosynthesis.

## Author contributions

GCV planned the research; KD and GCV planned and designed the experiments; KD, GDM and NAA performed the experiments; KD and GCV analyzed the data; and GCV wrote the paper.

## Supplementary Material

Supplementary Data

## Abbreviations:

A: net CO_2_ assimilation rate
AOX: alternative oxidase
EP: excitation pressure
ETC: electron transport chain
ETR: linear electron transport rate
*g*_s_: stomatal conductance
LHCB2: light-harvesting complex II protein
NPQ: non-photochemical quenching
PPFD: photosynthetic photon flux density
PSII: photosystem II
Φ_PSII_: effective quantum yield of PSII
PQ: plastoquinone
qP: photochemical energy quenching
*R*_D_: respiration rate in the dark
*R*_L_: respiration rate in the light
RWC: relative water content
TCA: tricarboxylic acid
WT: wild-type.
