# Supplementary material for: Coordinated regulation of photosynthetic and respiratory components is necessary to maintain chloroplast energy balance in varied growth conditions
Source: J Exp Bot. 2016 Dec 23;68(3):657–71. doi: 10.1093/jxb/erw469 (PMC5441918; doi:10.1093/jxb/erw469)
Supplement: Supplementary Data [file erw469_Supplementary_Data.zip › supplementary_figures_S1_S6.pdf]

Coordinate regulation of photosynthetic and respiratory components necessary to maintain chloroplast energy balance in varied growth conditions

Supplementary Figures S1 to S6

Dahal *et al*.

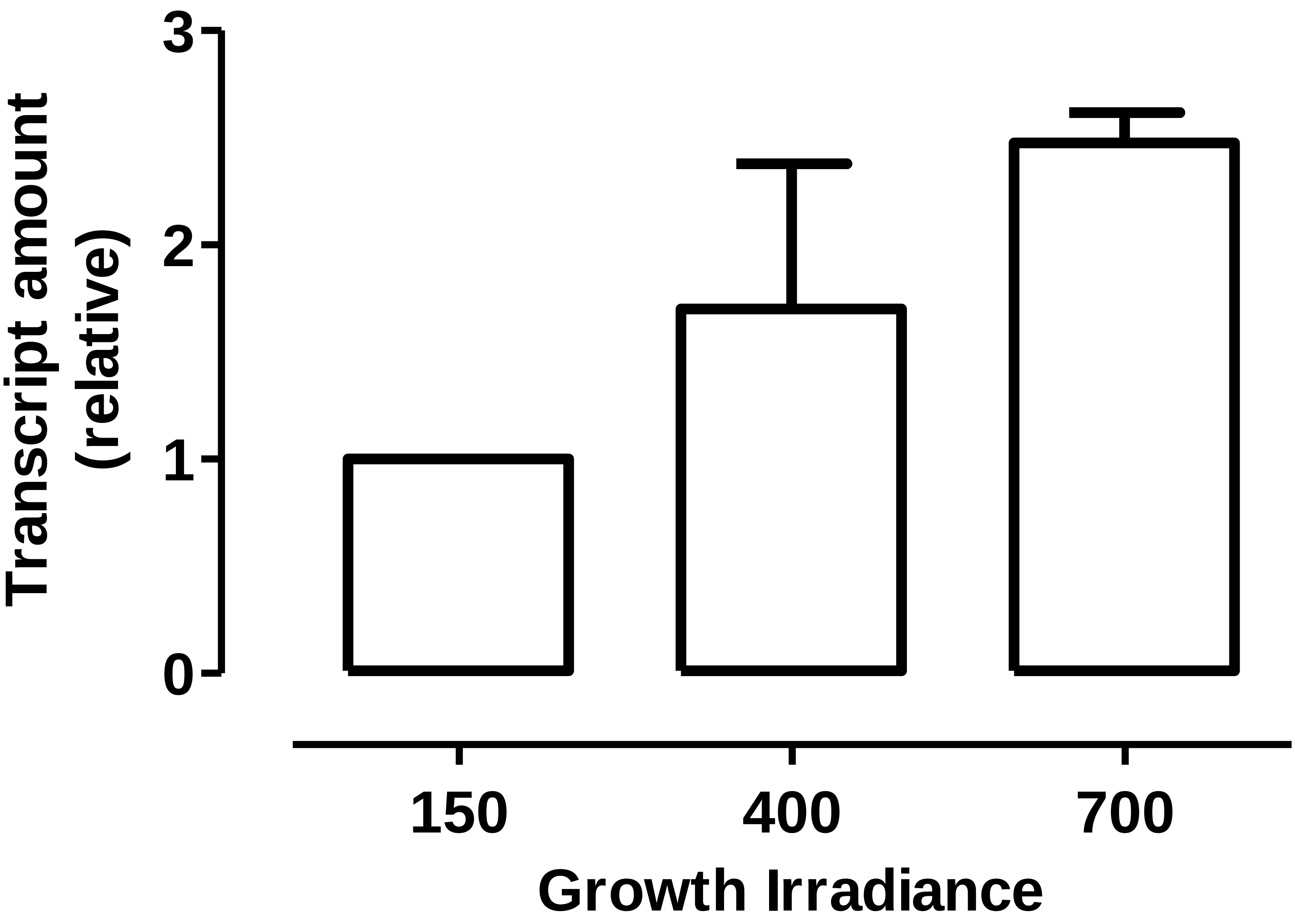

3
2
1
0
Transcript amount (relative)
150
400
700
Growth Irradiance

Figure S1

Supplementary Fig. S1. Relative *AOX1a* transcript amount in leaf of WT tobacco grown at 150, 400 or 700 PPFD under well-watered conditions for 19 to 21 days. Transcript amounts are relative to that of 150 PPFD-grown plants, which was set to 1. Data are the average +/- S.E. of three independent experiments.

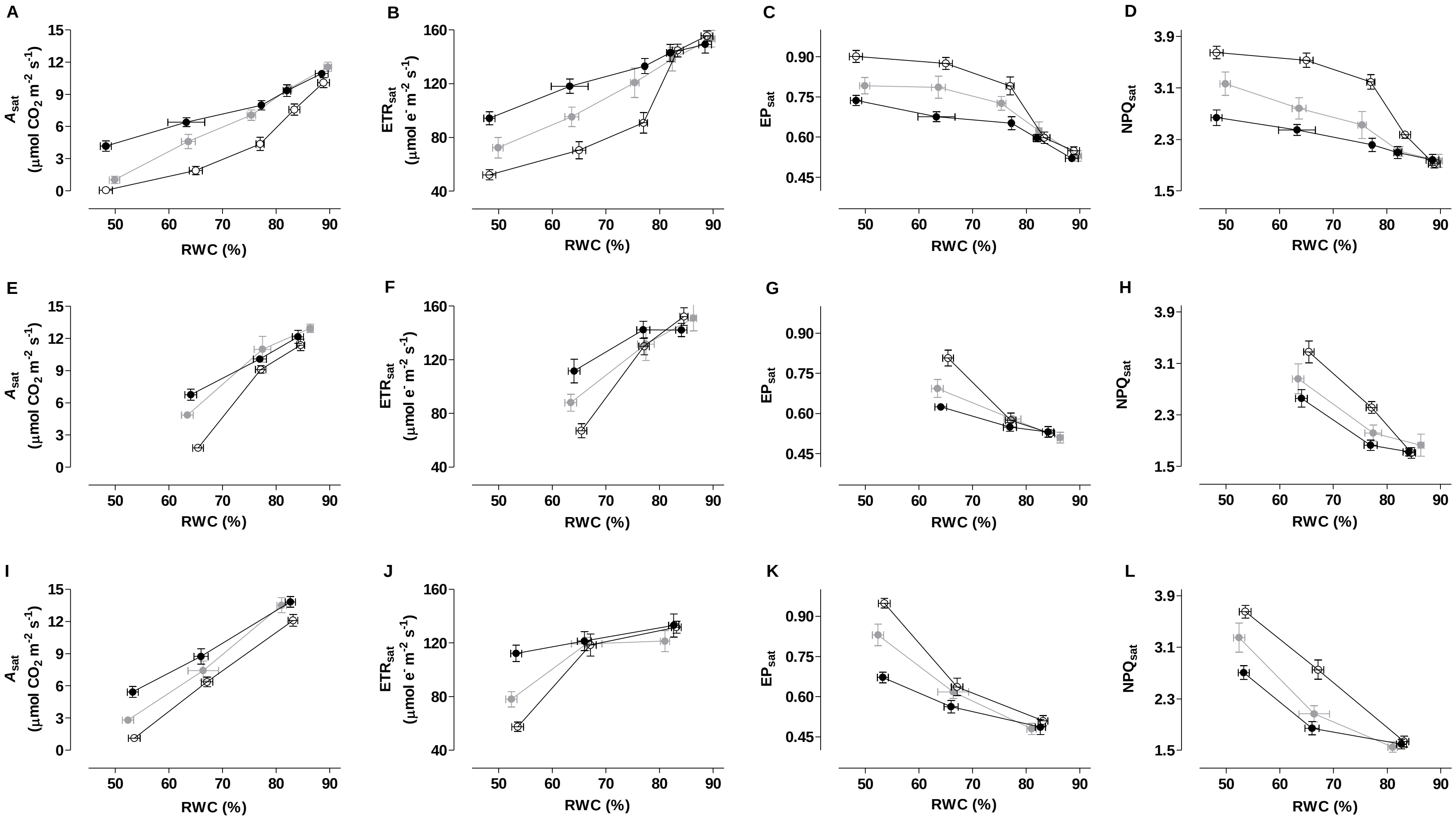
A
B
C
D
E
F
G
H
I
J
K
L
A_{sat} (μmol CO_2 m^{-2} s^{-1})
ETR_{sat} (μmol e^- m^{-2} s^{-1})
EP_{sat}
NPQ_{sat}
RWC (%)

Figure S2

Supplementary Fig. S2. Effect of growth irradiance and leaf water status on photosynthesis in WT tobacco and transgenic lines with altered amounts of AOX protein. (A,E,I) A_{sat}. (B,F,J) ETR_{sat}. (C,G,K) EP_{sat}. (D,H,L) NPQ_{sat}. Plants were grown at 150 PPFD (A-D), 400 PPFD (E-H) or 700 PPFD (I-L) under well-watered conditions for 19 to 21 days, followed by water being withheld from the plants for up to an additional 6 days. At different times following the water being withheld, each of the photosynthetic parameters was measured at saturating irradiance (1600 PPFD). Data are shown for WT plants (gray circles), AOX overexpressors (solid circles) and AOX knockdowns (open circles). In each independent experiment, data from two overexpressors (B7 and B8) that acted similarly were averaged, and data from two knockdowns (RI9 and RI29) that acted similarly were averaged. The data shown are the average +/- S.E. of three to five independent experiments.

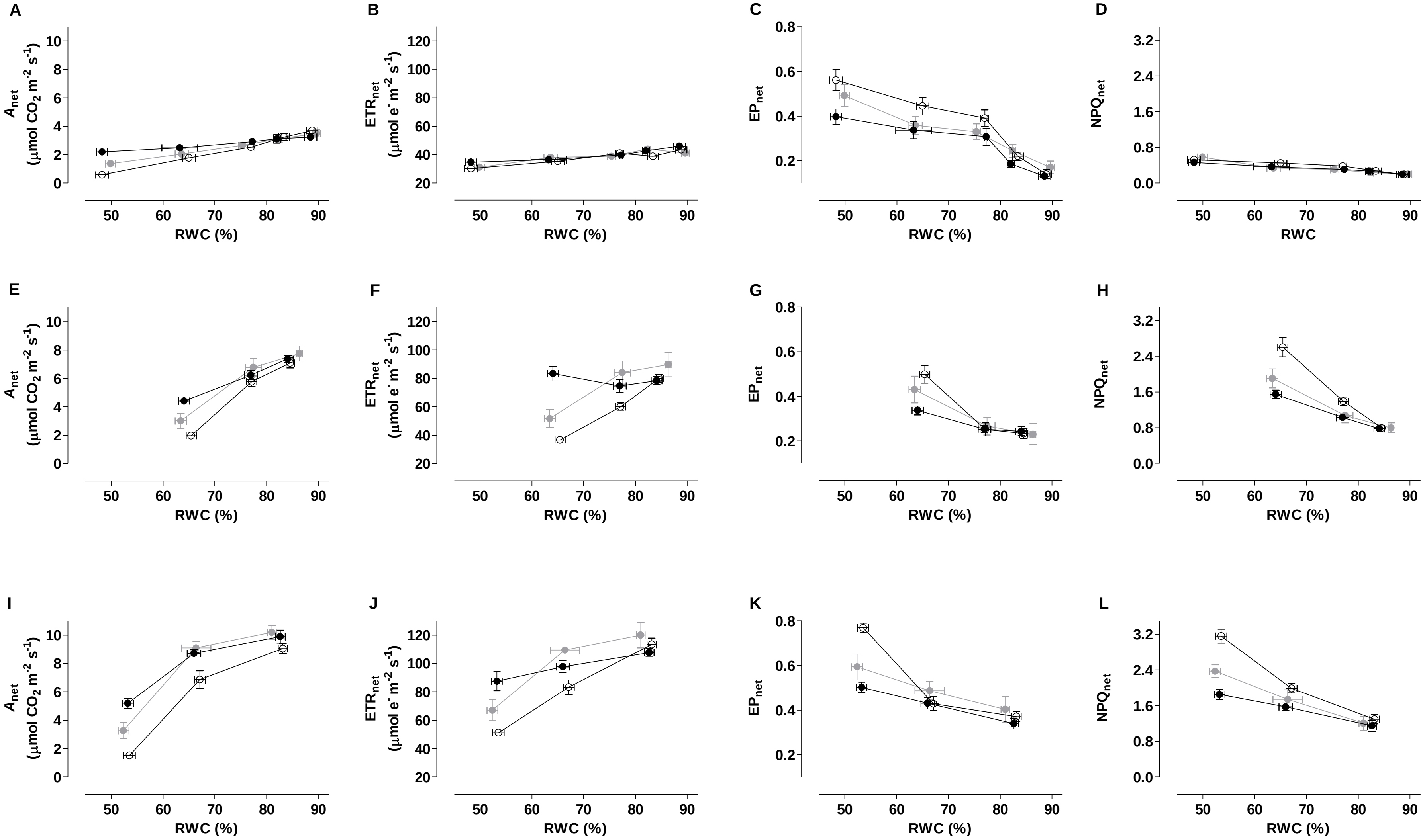

A
B
C
D
E
F
G
H
I
J
K
L
A_{net} (μmol CO_2 m^{-2} s^{-1})
ETR_{net} (μmol e^- m^{-2} s^{-1})
EP_{net}
NPQ_{net}
RWC (%)
RWC

Figure S3

Supplementary Fig. S3. Effect of growth irradiance and leaf water status on photosynthesis in WT tobacco and transgenic lines with altered amounts of AOX protein. (A,E,I) A_{net}. (B,F,J) ETR_{net}. (C,G,K) EP_{net}. (D,H,L) NPQ_{net}. Plants were grown at 150 PPFD (A-D), 400 PPFD (E-H) or 700 PPFD (I-L) under well-watered conditions for 19 to 21 days, followed by water being withheld from the plants for up to an additional 6 days. At different times following the water being withheld, each of the photosynthetic parameters was measured at the growth irradiance (150, 400 or 700 PPFD). Data are shown for WT plants (gray circles), AOX overexpressors (solid circles) and AOX knockdowns (open circles). In each independent experiment, data from two overexpressors (B7 and B8) that acted similarly were averaged, and data from two knockdowns (RI9 and RI29) that acted similarly were averaged. The data shown are the average +/- S.E. of three to five independent experiments.

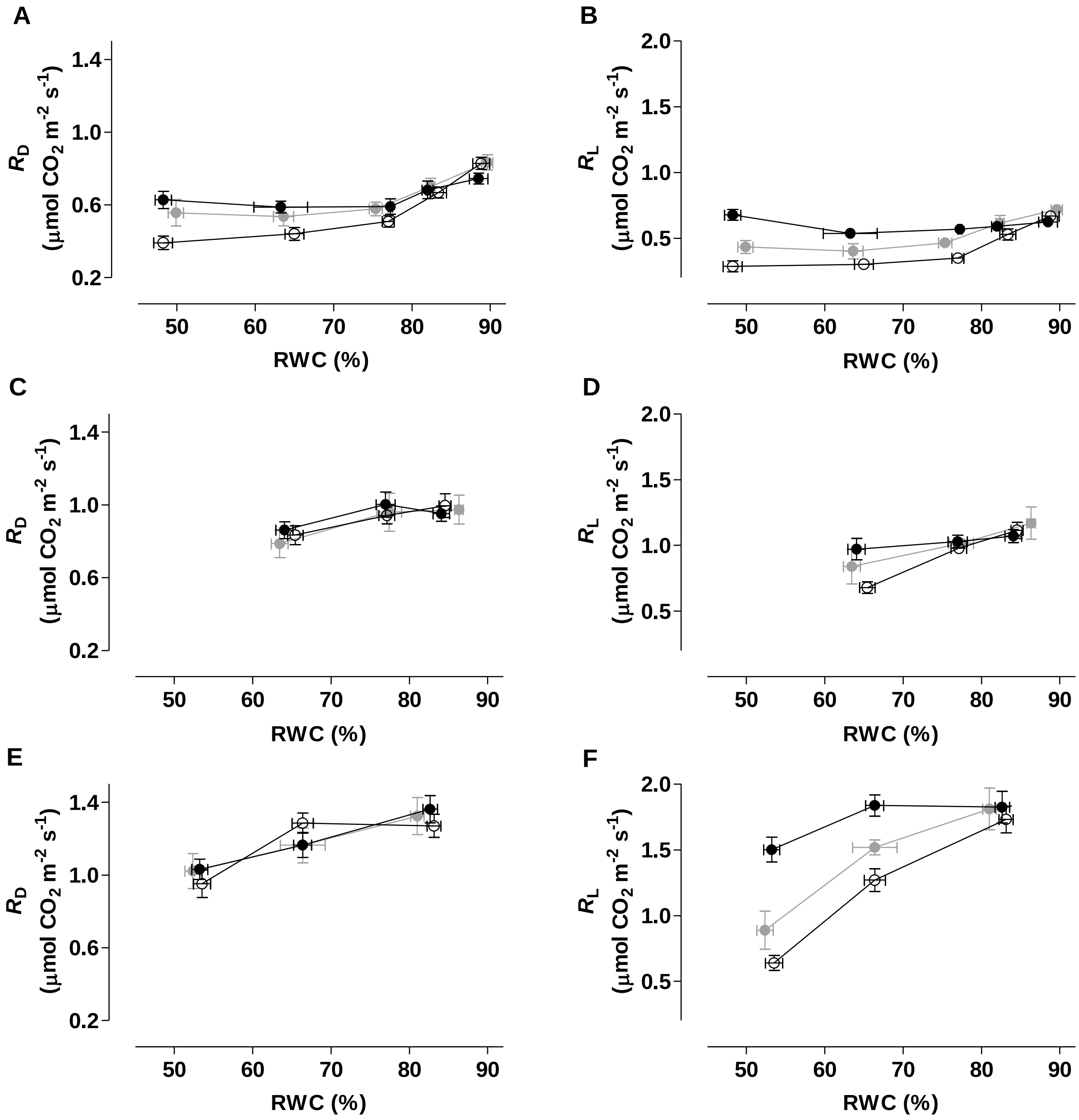

Figure S4

Supplementary Fig. S4. Effect of growth irradiance and leaf water status on respiration in WT tobacco and transgenic lines with altered amounts of AOX protein. (A,C,E) Leaf R_D. (B,D,F) Leaf R_L. Plants were grown at 150 PPFD (A,B), 400 PPFD (C,D) or 700 PPFD (E,F) under well-watered conditions for 19 to 21 days, followed by water being withheld from the plants for up to an additional 6 days. At different times following the water being withheld, respiration and leaf RWC were determined. Data are shown for WT plants (gray circles), AOX overexpressors (solid circles) and AOX knockdowns (open circles). In each independent experiment, data from two overexpressors (B7 and B8) that acted similarly were averaged, and data from two knockdowns (RI9 and RI29) that acted similarly were averaged. The data shown are the average +/- S.E. of three to five independent experiments.

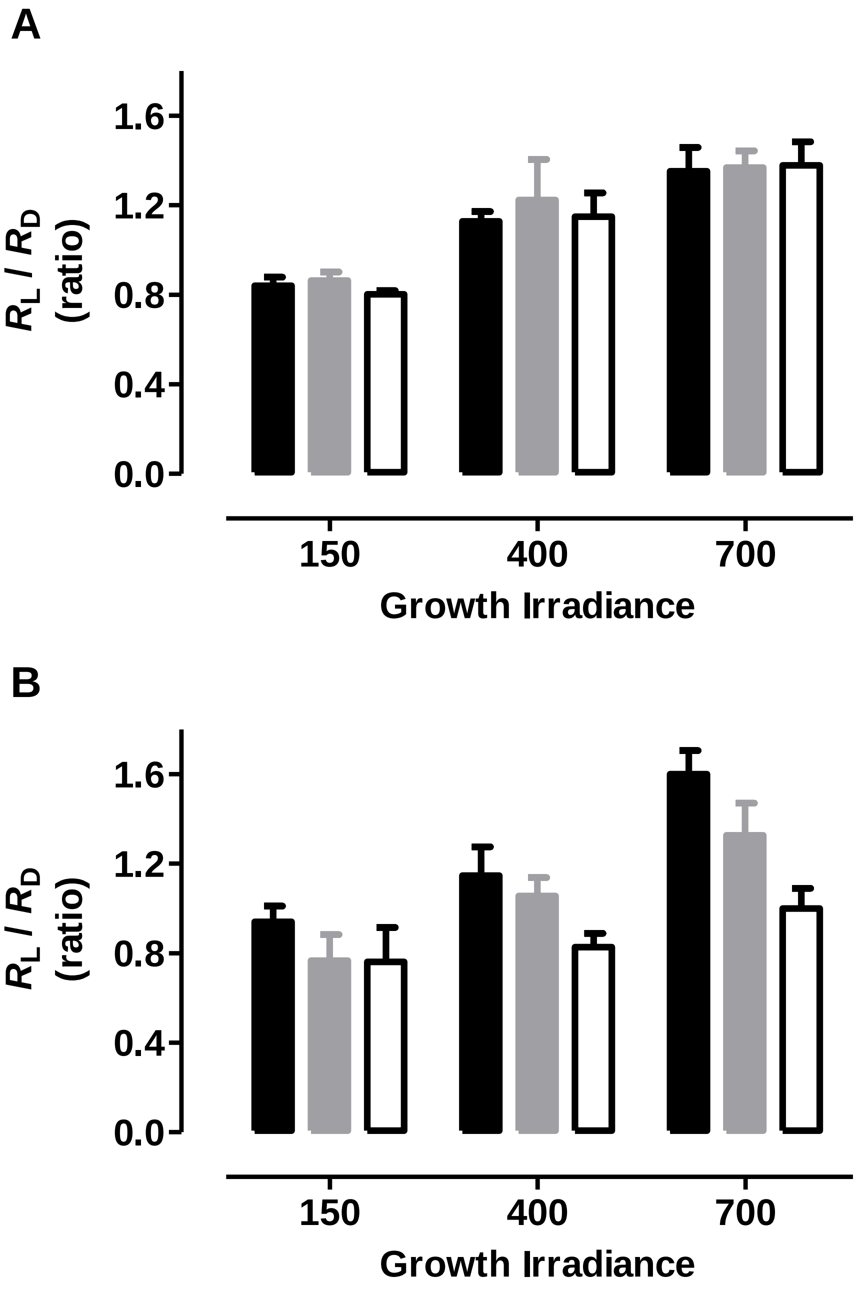

Figure S5

Supplementary Fig. S5. Effect of growth irradiance on the R_L/R_D ratio in well-watered tobacco plants (A) and plants experiencing moderate drought (leaf RWC of 63-66%). Data are shown for WT plants (gray bars), AOX overexpressors (solid bars) and AOX knockdowns (open bars). In each independent experiment, data from two overexpressors (B7 and B8) that acted similarly were averaged, and data from two knockdowns (RI9 and RI29) that acted similarly were averaged. The data shown are the average +/- S.E. of three to five independent experiments.

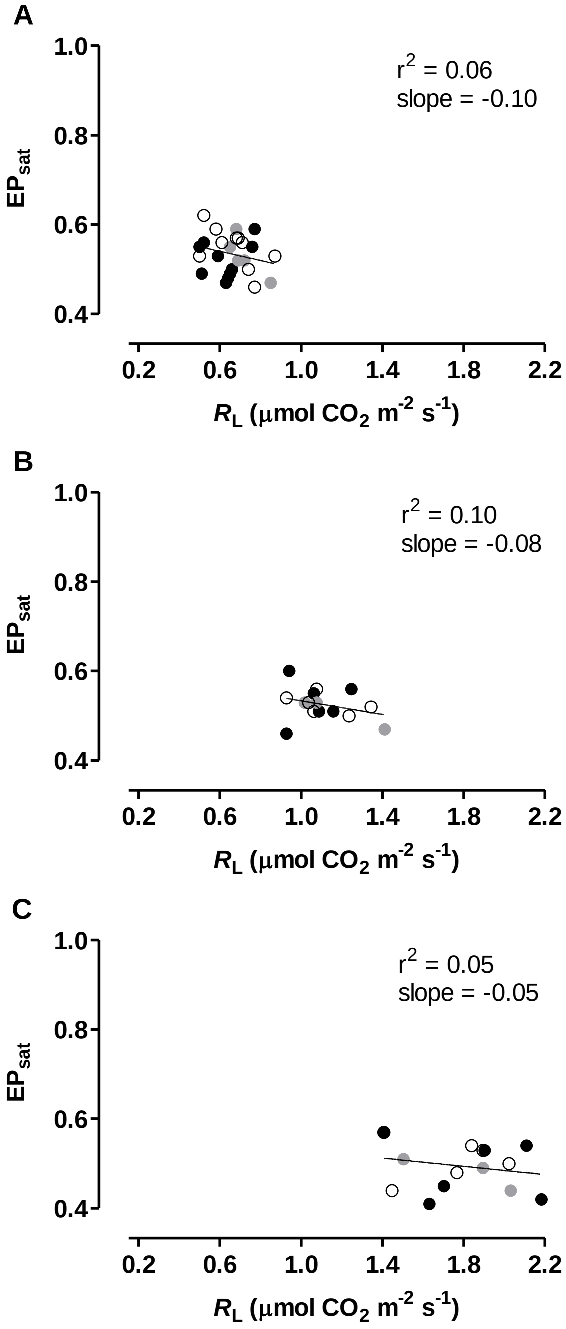
A
r2 = 0.06
slope = -0.10
EPsat
RL (µmol CO2 m-2 s-1)
B
r2 = 0.10
slope = -0.08
C
r2 = 0.05
slope = -0.05

Figure S6

Supplementary Fig. S6. Changes in R_L and EP_{sat} as a function of growth irradiance in well-watered WT tobacco and transgenic lines with altered amounts of AOX protein. Plants were grown at 150 PPFD (A), 400 PPFD (B) or 700 PPFD (C) under well-watered conditions for 19 to 21 days. Leaf R_L and EP_{sat} were then determined. Data are shown for WT plants (gray circles), AOX overexpressors (solid circles; half are B7, half are B8) and AOX knockdowns (open circles; half are RI9, half are RI29). The data points are compiled from 3 to 5 independent experiments.
